# Lung and gut microbiomes in pulmonary aspergillosis: Exploring adjunctive therapies to combat the disease

**DOI:** 10.3389/fimmu.2022.988708

**Published:** 2022-08-12

**Authors:** Liuyang Cai, Peigen Gao, Zeyu Wang, Chenyang Dai, Ye Ning, Macit Ilkit, Xiaochun Xue, Jinzhou Xiao, Chang Chen

**Affiliations:** ^1^ Shanghai Engineering Research Center of Lung Transplantation, Shanghai, China; ^2^ Basic School of Medicine, Second Military Medical University (Naval Medical University), Shanghai, China; ^3^ Department of Thoracic Surgery, Shanghai Pulmonary Hospital, Tongji University School of Medicine, Shanghai, China; ^4^ Division of Mycology, Department of Microbiology, Faculty of Medicine, University of Çukurova, Adana, Turkey; ^5^ Department of Pharmacy, 905th Hospital of People’s Liberation Army of China (PLA) Navy, Shanghai, China; ^6^ Shanghai Engineering Research Center of Hadal Science and Technology, College of Marine Sciences, Shanghai Ocean University, Shanghai, China

**Keywords:** pulmonary aspergillosis, fungal diseases, *Aspergillus*, immunity, microbiome

## Abstract

Species within the *Aspergillus* spp. cause a wide range of infections in humans, including invasive pulmonary aspergillosis, chronic pulmonary aspergillosis, and allergic bronchopulmonary aspergillosis, and are associated with high mortality rates. The incidence of pulmonary aspergillosis (PA) is on the rise, and the emergence of triazole-resistant *Aspergillus* spp. isolates, especially *Aspergillus fumigatus*, limits the efficacy of mold-active triazoles. Therefore, host-directed and novel adjunctive therapies are required to more effectively combat PA. In this review, we focus on PA from a microbiome perspective. We provide a general overview of the effects of the lung and gut microbiomes on the growth of *Aspergillus* spp. and host immunity. We highlight the potential of the microbiome as a therapeutic target for PA.

## Introduction

**Figure 1:**
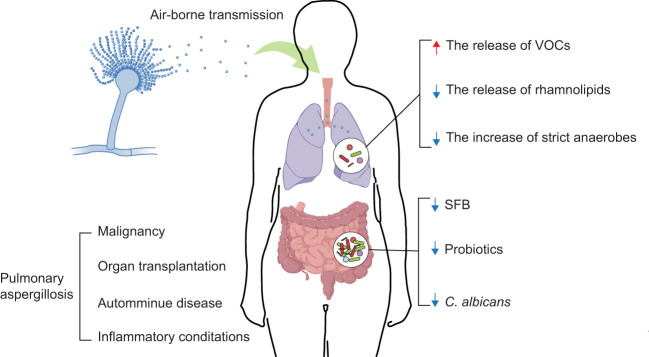
Interaction between the microbiome and PA. *Aspergillus* spp. conidia usually enter the human body through air-borne transmission. The colonization and infection of *Aspergillus* spp. have dramatically increased, considering the growing numbers of patients with an impaired immune state associated with the treatment of malignancy, organ transplantation, autoimmune diseases, and inflammatory conditions (4, 5). The development of PA is influenced by composition and metabolites of the lung and gut microbiomes (e.g., VOCs, rhamnolipids, strict anaerobes, SFB, probiotics, *C. albicans*). ↑: increase; ↓: decrease.

Pulmonary aspergillosis (PA) is an infection or allergic response caused by *Aspergillus* spp (1). *Aspergillus* spp. are widely present in the environment and are mainly transmitted via airborne conidia (2, 3) (**Figure 1**). *Aspergillus fumigatus* is one of the most common *Aspergillus* spp. It is responsible for the majority of PA incidences (6). Depending on host immunity, pulmonary ailments caused by *Aspergillus* spp. can be mainly classified as invasive pulmonary aspergillosis (IPA), chronic pulmonary aspergillosis (CPA), or allergic bronchopulmonary aspergillosis (ABPA) (6, 7). Recent global estimates revealed that 8,000,000 cases of PA occur annually (8). Mold-active triazole exerts cidal activity, and as such are the frontline antifungals used to treat aspergillosis, while echinocandins show static activity and amphotericin B prescription is limited owing to its cytotoxic activity (9). Unfortunately, the extensive use of fungicides in the environment as well as in the clinic, has resulted in the increasing emergence of triazole resistant aspergillosis, mostly owing to *A*. *fumigatus* (9–12). Additionally, expensive and/or toxic drugs, drug-drug interactions, and unequal clinical resources in different regions reduce the potential for survival and recovery (13, 14). Therefore, there is an unmet need to identify/design novel antifungal drugs as well as make use of host-directed strategies to combat PA and to improve the clinical outcomes of inflicted patients.

Despite being a novel field, the current paradigm suggests that communities of microbes living on various epithelial surfaces, known as microbiome, are linked to an array of complications in humans and dysregulation in the composition of such communities, known as microbiome dysbiosis, can have profound impact on predisposition to various infections and complications ranging from pulmonary infections and cancer to diabetes and neurological disorders (15–19). On the one hand, the application of fecal microbial transplantation from healthy donors to patients was found to be promising against a wide range of ailments. On the other hand, human complications are often accompanied by microbiome alterations, and as such, determination of the microbiome signature could potentially offer a robust diagnostic tool, and its leverage could subsequently aid in timely and effective treatment. For instance, Hérivaux et al. (20) reported that microbiome diversity was found to predict IPA onset and the mortality rate associated with this complication. Apart from scattered studies reported thus far, the association of the human microbiome with PA remains largely elusive. Determination of a clear picture of the healthy microbiome and dysbiosis in the context of PA could potentially enhance the therapeutic capacity; therefore, the current study thoroughly discusses and links the human microbiome to PA.

Although the microbiome composition of the lung remained elusive in early times, the development of quantitative molecular sequencing methods has identified a complicated microbial community inhabiting the lung, known as the lung microbiome (21). The lung microbiome is associated with immune activation and regulation (22); it is also known to diverge substantially between healthy (23) and diseased states (24, 25). Dysbiosis of the lung microbiome is related to the exacerbations of several respiratory diseases such as bronchiectasis, cystic fibrosis (CF), and chronic obstructive pulmonary disease (26–28). The components and metabolites of the gut microbiome can also influence immune responses (29). Intestinal dysbiosis has been linked to alterations in host immunity and disease development, including respiratory diseases (30). Moreover, numerous pieces of evidence support the key contribution of the microbiome in the prevention and treatment of respiratory diseases. Gram-negative bacilli (*Pseudomonas aeruginosa*, *Acinetobacter baumannii*, and *Escherichia coli*, etc.) that colonize the lungs usually cause nosocomial pneumonia. Antimicrobial therapy improves the outcome of nosocomial pneumonia (31). Gut commensal microbiome regulate immune responses in the respiratory mucosa and resist respiratory virus infections (32). Although the significance of the microbiome has already been established in respiratory diseases, the mechanisms by which the lung and gut microbiomes influence PA are relatively unknown (33, 34). Considering this, in this review, we aim to discuss the role of the lung and gut microbiomes in the growth of *Aspergillus* spp. and host immunity (**Figure 1**, **Table 1**). We hope it will serve as a vital foundation for the further analysis of the interactions between immunity, the microbiome, and PA. Moreover, our review will contribute to the development of a more reliable clinical treatment for PA.

**Table 1:** The possible mechanisms underlying the effects of the lung and gut microbiomes on PA.

Microbial colonization site	Alterations in the microbiome	Possible mechanism	References
Lung	*P. aeruginosa* or other gram-negative bacteria	Release of VOCs to stimulate the growth of *A*. *fumigatus* without direct contact.	(35, 36)
	*P. aeruginosa*	Release of rhamnolipids to inhibit the growth of *A*. *fumigatus* by direct contact.	(37)
Gut	The variety of bacteria declines, whereas fungi increase quickly	Worsen the prognosis of IPA.	(20)
	The increase of strict anaerobes	Reduces the risk of PA by limiting the expansion of pathogenic *Proteobacteria*.	(38, 39)
	SFB Probiotics	Stimulate lung autoimmunity by inducing IL-1 receptor ligands and Th17 cells. Affect immune cells or release metabolites to inhibit the development of PA.	(40–42) (43–47)
	Gut microbiome disrupted by antibiotics	The overgrowth of *C. albicans* induces cross-reactive Th17 cells to promote ABPA.	(48–51)

VOCs, volatile bacterial organic compounds; IPA, invasive pulmonary aspergillosis; SFB, segmented filamentous bacteria; PA, pulmonary aspergillosis; ABPA, allergic bronchopulmonary aspergillosis.

## Advances in immunity to pulmonary aspergillosis

Conventionally, a few members of the *Aspergillus* spp. have reached the alveoli and exposed the cell wall pathogen-associated molecular patterns, such as β-D-glucan (52, 53). In immunocompetent individuals, different pattern recognition receptors (PRRs) include Toll-like receptors, C-type lectin receptors (CLRs), and Nod-like receptors. PRRs can recognize *Aspergillus* spp. and initiate an early immune response (54). For instance, Dectin-1, a CLR, recognizes fungal β-glucan and modulates the inflammatory responses by inducing the expression of the anti-inflammatory cytokine interleukin (IL)-10 (55). Subsequently, innate immune cells (macrophages, neutrophils, etc.) actively participate in the cellular immune responses against *Aspergillus* spp. by engulfing and killing the conidia. As the main resident leukocytes in the lungs, alveolar macrophages can rapidly adhere to and take up conidia that enter the alveolar space (56). In contrast to delayed killing mediated by alveolar macrophages, neutrophilic granulocytes rapidly kill hyphae of *Aspergillus* spp. through an active oxygen-dependent mechanism at the cell surface (57). These innate immune responses constitute the first line of defense against pulmonary host defense and the natural and chemical barriers of the organism.

Nonetheless, for patients with chronic respiratory disease or impaired immune function (e.g., neutropenia), these innate immune responses do not function normally leading to *Aspergillus* spp. colonization and infection. In this case, the adaptive immune responses are activated. CD4 (including Th1, Th2, Th17, etc.) or CD8 T-cell responses play a critical role in PA. After infection with *Aspergillus* spp., Th1 cells enhance the antifungal activities of macrophages and neutrophils and express the pro-inflammatory cytokines TNF-α and IFN-γ (58). Conversely, Th2 cell activation inhibits Th1 cell responses. Allard et al. (59) reported a direct airway exposure to *Aspergillus* spp. Lysates boost the Th2 cell responses in the lungs of mice, resulting in symptoms similar to those of ABPA. Symptoms include eosinophilic inflammation, mucus hypersecretion, and increased airway resistance. In contrast, the role of Th17 cell responses in *Aspergillus* spp. infection is debatable. IL-17 and IL-23 produced by Th17 cells can suppress Th1-mediated protective immunity against fungi and increase susceptibility to *Aspergillus* spp. in mice (60). However, some studies have concluded that IL-17 is involved in protective responses against PA. For example, Werner et al. (61) observed that the neutralization of IL-17 significantly impaired *A*. *fumigatus* clearance. In summary, innate and adaptive immune responses help host resistance against PA.

Relevant advances have been made in devising immunotherapeutic strategies for PA. Among the innate immune responses, Bruton’s tyrosine kinase (BTK), a key molecule in multiple signaling pathways, activates fungal recognition immune responses. The clinical application of BTK inhibitors is to impair several immune functions of platelets in response to *A*. *fumigatus* and increases the risk of invasive aspergillosis in patients with chronic lymphocytic leukemia (62). Among the adaptive immune responses, from a neutropenia perspective, transfusable neutrophil progenitors serve as new cellular therapies for the prevention of IPA. This treatment produces unlimited numbers of homogenous granulocyte-macrophage progenitors, greatly improving survival in models of PA (63). Although an increasing number of new therapeutic strategies are being discovered, most of the studies are still limited to animal experiments. Whether these therapeutic strategies are applicable to humans remains uncertain and cannot be extrapolated directly. Thus, further studies are needed to determine how the immune system functions during *Aspergillus* spp. infection. Researchers should strive to translate these findings into valuable therapeutic tools for clinical settings.

## Effects of the lung microbiome on pulmonary aspergillosis

Lung bacteria are vital in protecting against PA. *Pseudomonas aeruginosa* and *A*. *fumigatus* frequently coexist in the lungs. These two species have competitive interactions that can influence the growth of the microbiome and disease outcomes. Volatile bacterial organic compounds (VOCs) produced by *P*. *aeruginosa* or other gram-negative bacteria (e.g., *E*. *coli* and *Burkholderia cepacia*) can stimulate the growth of *A*. *fumigatus* without direct contact (35, 36) (**Figure 1**, **Table 1**). Li et al. (64) identified VOCs that can be used as biomarkers for differential diagnosis and therapeutic response prediction in patients with CPA. In contrast, *A*. *fumigatus* biofilm formation is inhibited by direct contact with *P*. *aeruginosa* (65). *Pseudomonas aeruginosa* showed a strong association with *A*. *fumigatus* hyphae. When *P*. *aeruginosa* is in direct contact with *A*. *fumigatus*, the diffusible extracellular molecules produced by *P*. *aeruginosa* disrupt its growth. Specifically, rhamnolipids secreted by *P*. *aeruginosa* block fungal β1,3 glucan synthase activity. Rhamnolipids inhibit the growth of *A*. *fumigatus* in *in vitro* experiments (37) (**Figure 1**, **Table 1**). Moreover, Hérivaux et al. (20) observed a loss of bacterial diversity and overgrowth of bacteria (e.g., *Staphylococcus*, *Escherichia*, *Paraclostridium*, and *Finegoldia* genera) in the lungs of patients with IPA. These changes in the lung microbiome were predictive of disease outcomes across IPA. In summary, there were complex reactions between lung bacteria and *A*. *fumigatus*. The growth of *A*. *fumigatus* may be regulated by lung bacteria, which, in turn, affects the severity of PA. Most of the studies were conducted in the context of CF, which has some similarities to the regulation of *A*. *fumigatus* growth by lung bacteria during PA; however, further validation is needed.

However, little is known about the direct regulation of PA by lung fungi. Several studies have focused on fungi that interact with lung bacteria and indirectly influence PA. *Candida albicans* colonization of the airway increases the prevalence of *P*. *aeruginosa* in rat lungs by inhibiting the production of reactive oxygen species by alveolar macrophages (66). An increase in the prevalence of *P*. *aeruginosa* in the lungs is likely to accelerate the growth of *A*. *fumigatus* and induce PA. Additionally, some studies have shown that changes in the composition of the lung microbiome can predict the survival of patients with IPA. On the one hand, the variety of lung bacteria declines, whereas lung fungi increase quickly. These changes worsen the prognosis of patients with IPA (20) (**Table 1**). On the other hand, the increase in strict anaerobes in the lungs reduces the risk of *A*. *fumigatus* infection by limiting the expansion of pathogenic *Proteobacteria* (38, 39) (**Figure 1**, **Table 1**). Notably, the lung microbiome has been shown to play a role in the regulation of *A*. *fumigatus* growth and even influence the progression of PA. However, studies on how the lung microbiome affects PA remain inadequate. As a potential treatment for PA, there is immense potential for future research on the lung microbiome.

The role of the lung microbiome in PA is probably largely underestimated because of non-specific and insensitive sampling and diagnostic tools. Compared with the gut microbiome, the lung microbiome is not easy to obtain and has low microbial biomass (67). Owing to the existence of physiological processes (e.g., aspiration), it is difficult to avoid the oral microbiome when trying to isolate the lung microbiome (23, 68). Deep sputum conjoint culture has been shown to distinguish oropharyngeal flora from lung fungi and diagnose lung fungal infections early (69). Pragman et al. (68) concluded that the lung lobectomy protocol utilized is well suited for obtaining reasonable non-invasive samples. Among the several methods used to obtain lung microbiome samples, bronchoscopy may cause sample contamination, but its effects are largely negligible (23). Micro-anatomical differences exist in the lung microbiome. Different parts of the lungs of the same individual have different microbiomes (70). In addition, individuals from different regions can also contain different lung microbiomes (71, 72). Rapid advances in technology have helped advance the study of the lung microbiome; however, several related problems exist owing to the lack of standardization. Diagnostic tools include high-throughput sequencing, phylogenetic microarray analysis, terminal restriction fragment length polymorphism, and amplicon length heterogeneity-polymerase chain reaction. The results obtained by different analytical methods vary (73, 74). In conclusion, many studies on the lung microbiome have been limited by small sample sizes, different sample collection techniques, different sampling sites, different regions of the subjects, and different analysis techniques. These limitations make it difficult to compare the results of the different studies. The impact of the lung microbiome on PA is an emerging area that needs further exploration and validation. Moreover, to better assess different studies and acquire reliable findings, continued research in this field is likely to establish a standardized method for obtaining and analysing the lung microbiome.

## Role of the gut microbiome in pulmonary aspergillosis

Bacteria colonizing the intestinal mucosa are involved in the maintenance of host immune homeostasis. Normal immune homeostasis further helps the host remove invasive fungi from the outside world. Ivanov et al. (40) found that segmented filamentous bacteria (SFB) can colonize the surface of the ileum in mice and induce intestinal CD4(+) T helper cells to produce IL-17 and IL-22 (Th17 cells). Furthermore, during fungal infections, SFB can induce lung autoimmunity by stimulating the systemic release of IL-1 receptor ligands and inducing gut-lung axis Th17 cells expressing dual TCR (41, 42) (**Figure 1**, **Table 1**). Mice infected with *A*. *fumigatus* show changes in the diversity of their gut bacteria, which affects intestinal immune tolerance and predisposes them to intestinal inflammation (75).

Probiotics are promising new targets for antifungal treatments. Probiotics can influence the constituents of the gut microbiome by directly affecting immune cells or releasing health-promoting metabolites, which, in turn, affects systemic immunity (43) (**Figure 1**, **Table 1**). For example, oral treatment with live *Lactobacillus reuteri* and *Bifidobacterium longum* reduces allergic airway reactions (e.g., ABPA) by increasing the number of Tregs in the lungs (44, 45). Oral administration of bacteria expressing high levels of α-Gal can protect turkeys against an infectious challenge with *A*. *fumigatus* by reducing the levels of lung anti-α-Gal IgA (46). Despite the lack of oral experiments, the *E*. *coli* DH5α strain also inhibited the development of *A*. *fumigatus* conidia in *in vitro* experiments (47). In summary, understanding the interaction between gut bacteria and host immunity provides a potential therapeutic strategy for the treatment of PA.

Studies have revealed the existence of cross-protective immunity between *A*. *fumigatus* and *C*. *albicans*. The gastrointestinal system of mice treated with *C*. *albicans* was protected against IPA and vice versa. In addition, cross-protection between *A*. *fumigatus* and *C*. *albicans* is mediated by Th1 immunity and dependent on IFN-γ. IFN-γ-deficient mice vaccinated with *A*. *fumigatus* or *C*. *albicans* show no reduced fungal growth in the lungs or the gastrointestinal system, respectively (76). Noverr et al. (48–50) demonstrated that antibiotic treatment changed the composition of the gut microbiome, causing overgrowth of intestinal bacteria and *C*. *albicans* in mice. As a result, *Aspergillus*-infected mice were more sensitive to CD4 T cell-mediated pulmonary allergic airway responses (e.g., ABPA). The reason for this may be that the overgrowth of *C*. *albicans* increases plasma concentrations of prostaglandin E2 (PGE2) and induces M2 macrophage polarization in the lungs (77). PGE2 is required for the Th17 response, and *C*. *albicans* is the major fungal inducer of human Th17 responses (51, 78). Global antifungal Th17 modulation by *C*. *albicans* promotes pathogenic airway inflammation triggered by *A*. *fumigatus* in susceptible patients *via* the selective recruitment of cross-reactive Th17 cells (51) (**Figure 1**, **Table 1**). Particularly, even if mice are exposed to *Aspergillus* spp., allergic reactions will not occur in the airways if the gut microbiome is not damaged by antibiotics (49, 50). Advances in the understanding of the relationship between the increase in intestinal *C*. *albicans* and the occurrence of ABPA have highlighted the importance of gut fungi in maintaining host immunity and resistance to *Aspergillus* spp. Nevertheless, there is still some confusion and defects in the mechanisms by which gut fungi regulate pulmonary immunity after infection with *Aspergillus* spp. This should be further explored in the future and should not be limited to *C*. *albicans*.

## Discussion

The microbiome plays an important role in the prevention of PA by inhibiting the growth of *Aspergillus* spp. or by increasing host immunity. However, this new research field poses several technical challenges and unanswered questions. Thus far, there is a lack of standardized sampling of the lungs and uniform sequencing techniques for the identification of the lung microbiome (79). Subsequently, many questions remain unanswered regarding the interaction of the lung microbiome with the gut microbiome during *Aspergillus* spp. infection (80). The influence of the lung and gut microbiomes on healthy and immunocompromised individuals during *Aspergillus* spp. infection remains unclear (81). Further studies focused on these issues will contribute to a better understanding of the effect of the microbiome and immune system on PA. The development of PA will lead to the development of novel treatment strategies.

## Author contributions

XX, JX, and CC conceptualized the ideas. LC, PG, and ZW performed the literature search. LC wrote the original manuscript. CD, YN, and MI revised the manuscript. All authors contributed to the article and approved the submitted version.

## Funding

This study was supported by the scientific and technological innovation action plan of Science and Technology Commission of Shanghai Municipality (No. 20DZ2253700) and Shanghai Hospital Development Center (SHDC2020CR5012).

## Conflict of interest

The authors declare that the research was conducted in the absence of any commercial or financial relationships that could be construed as a potential conflict of interest.

## Publisher’s note

All claims expressed in this article are solely those of the authors and do not necessarily represent those of their affiliated organizations, or those of the publisher, the editors and the reviewers. Any product that may be evaluated in this article, or claim that may be made by its manufacturer, is not guaranteed or endorsed by the publisher.
